# Acute mesenteric ischemia secondary to metastatic neuroendocrine tumor: a case analysis and review of the literature

**DOI:** 10.1093/jscr/rjae725

**Published:** 2024-11-27

**Authors:** Emily P Swafford, Deepa R Magge

**Affiliations:** Department of Surgery, Vanderbilt University Medical Center, 1211 Medical Center Drive, Nashville, TN 37232, United States; Department of Surgery, Vanderbilt University Medical Center, 1211 Medical Center Drive, Nashville, TN 37232, United States

**Keywords:** neuroendocrine tumors, mesenteric ischemia, surgical oncology

## Abstract

Neuroendocrine tumors (NETs) are notably rare and frequently arise from the gastrointestinal tract. Generally asymptomatic, NETs uncommonly result in acute abdominal pain. We present a case of known metastatic NET manifesting as acute-on-chronic mesenteric ischemia due to the involvement of the superior mesenteric artery (SMA) and vein (SMV). A 63-year-old female with metastatic NET presented with acute-onset abdominal pain. The patient was hemodynamically stable but uncomfortable appearing with significant pain. Imaging demonstrated decreased enhancement of several small bowel loops within the right lower quadrant concerning for bowel ischemia with a mesenteric mass encasing the SMA and SMV. Surgical intervention revealed a nonviable loop of small bowel. Second-look laparotomy was performed with viable remaining bowel, and an ileocolic anastomosis was successfully created. Acute-onset abdominal pain in a patient with NET warrants urgent. Mesenteric ischemia, while rare, should not be overlooked, as timely diagnosis and intervention are imperative.

## Introduction

Neuroendocrine tumors (NETs), or tumors originating from neuroendocrine cells, are rare tumors with an incidence of only 9 per 100 000 [1]. NETs most frequently arise from the gastrointestinal tract, particularly the small bowel [2]. The majority of NETs are indolent in nature and grow slowly; however, some are more aggressive and present with later stage disease. About 10%–20% of patients with NETs present with metastatic disease at the time of diagnosis [3].

Given their location, small bowel neuroendocrine tumors (SBNETs) are often asymptomatic, allowing for a prolonged period of unrecognized growth. Symptoms, if present, most commonly manifest as non-specific abdominal pain. Delays in recognition may contribute to late-stage diagnosis [4]. Of patients with symptomatic SBNETs, only 25% to 35% present with acute symptoms with the most common presentation being intestinal obstruction (80%). Less common causes of acute-onset symptoms include pain due to mesenteric infiltration, intussusception, or mesenteric ischemia [5]. This study reports a rare case of metastatic SBNET manifesting as acute-on-chronic mesenteric ischemia due to the involvement of the superior mesenteric artery (SMA) and vein (SMV), necessitating urgent surgical intervention.

## Case report

A 63-year-old female with metastatic SBNET presented to the emergency department with acute-onset abdominal pain. Her symptoms began a few hours prior to presentation with abrupt, ten-out-of-ten abdominal pain. She had associated nausea and one episode of emesis. Notably, she had been experiencing less severe, postprandial abdominal pain over the last several months and decreased oral intake secondary to fear of pain.

**Figure 1:**
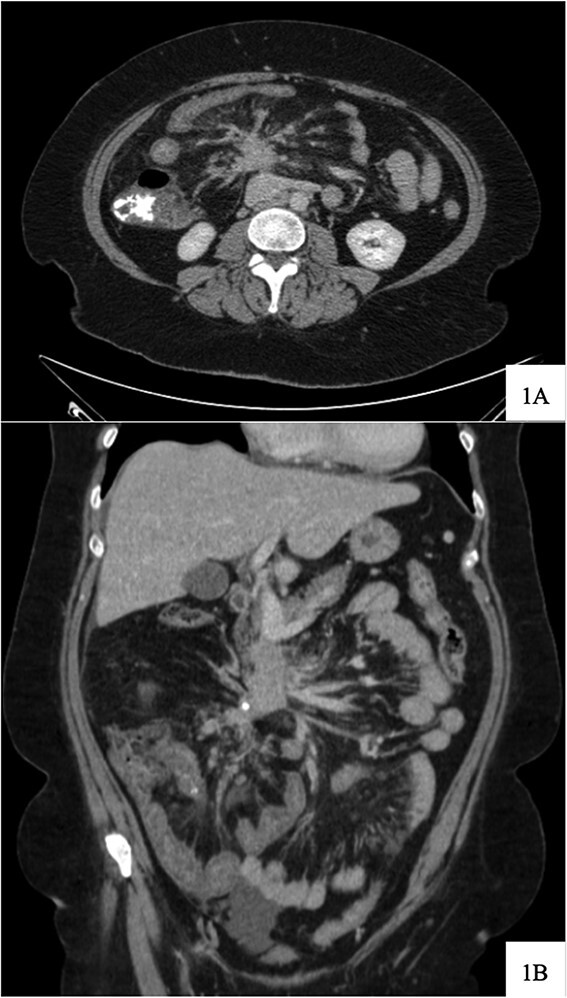
Computed tomography scan at time of diagnosis in September 2017 revealed a mass near the third portion of the duodenum and the root of the mesentery in the axial (A) and coronal (B) views.

**Figure 2:**
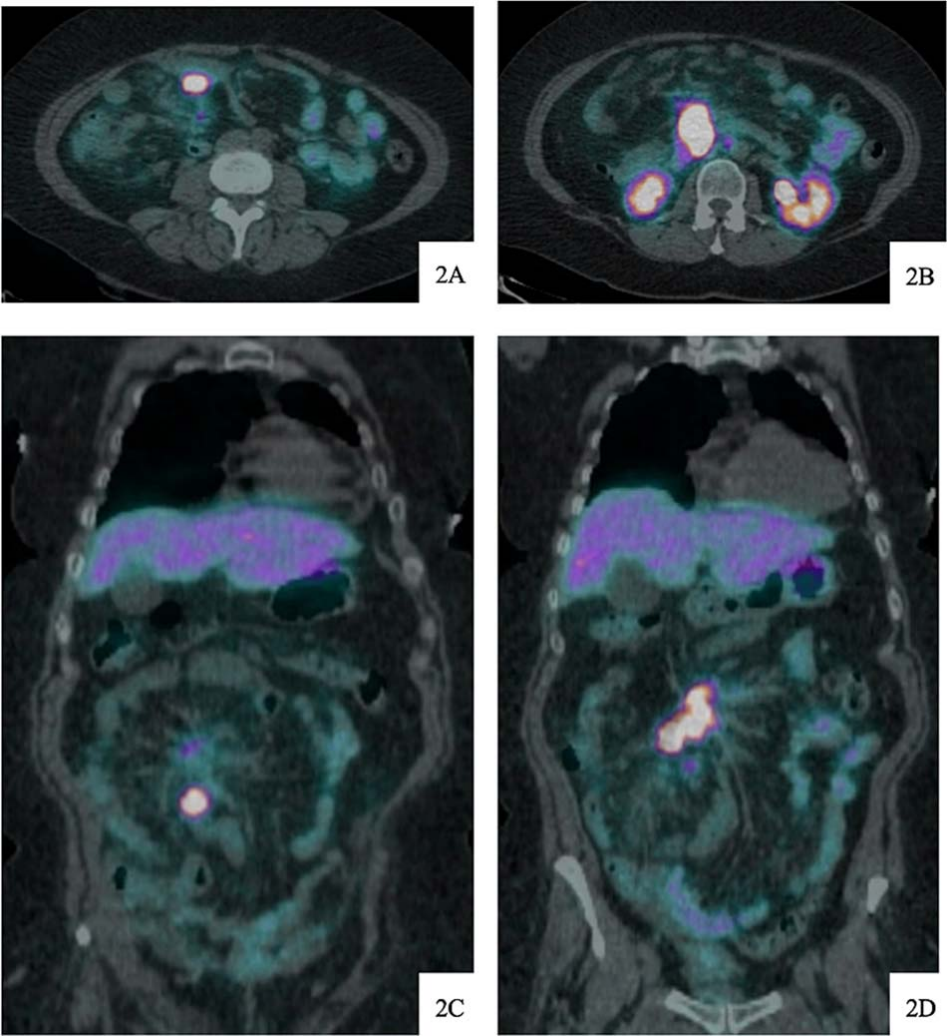
DOTATATE positron emission tomography-computed tomography scan at time of diagnosis in November 2017 revealed a small bowel primary tumor in the right lower quadrant consistent with terminal ileum (A) as well as the periduodenal mass near the root of the mesentery (B). Coronal reformatting redemonstrates the primary tumor (C) and mesenteric mass (D).

**Figure 3:**
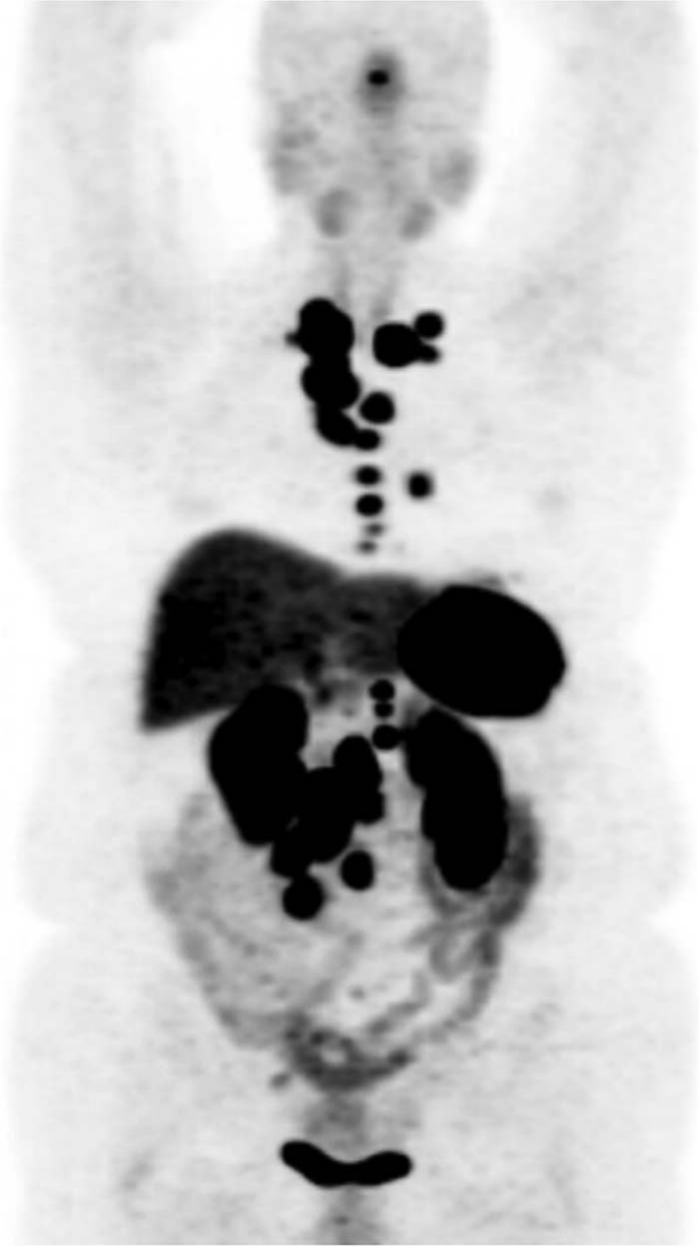
DOTATATE positron emission tomography-computed tomography scan revealing extent of metastatic disease.

Regarding her oncologic history, she was diagnosed with NET in October of 2017 when a computed tomography (CT) scan performed for intermittent abdominal cramping and diarrhea revealed a mass near the third portion of the duodenum and the root of the mesentery (Fig. 1). Biopsy of the mass revealed a well-differentiated NET. Further work-up with DOTATATE positron emission tomography (PET)/CT revealed a small bowel primary likely emanating from the terminal ileum as well as multiple lesions in the neck, chest, and liver (Figs 2 and 3). The periduodenal mass intimately involved the root of the mesentery, SMA, and SMV. She was initially treated with Lanreotide for many years with close monitoring and later underwent systemic treatment with Lutathera, a Peptide Receptor Radionuclide Therapy, from April 2020 to November 2020. She subsequently resumed Lanreotide in December of 2020, which she was on at the time of admission. Her disease remained largely stable on imaging over the next three years; however, her symptoms had been worsening over the few months leading up to her presentation.

**Figure 4:**
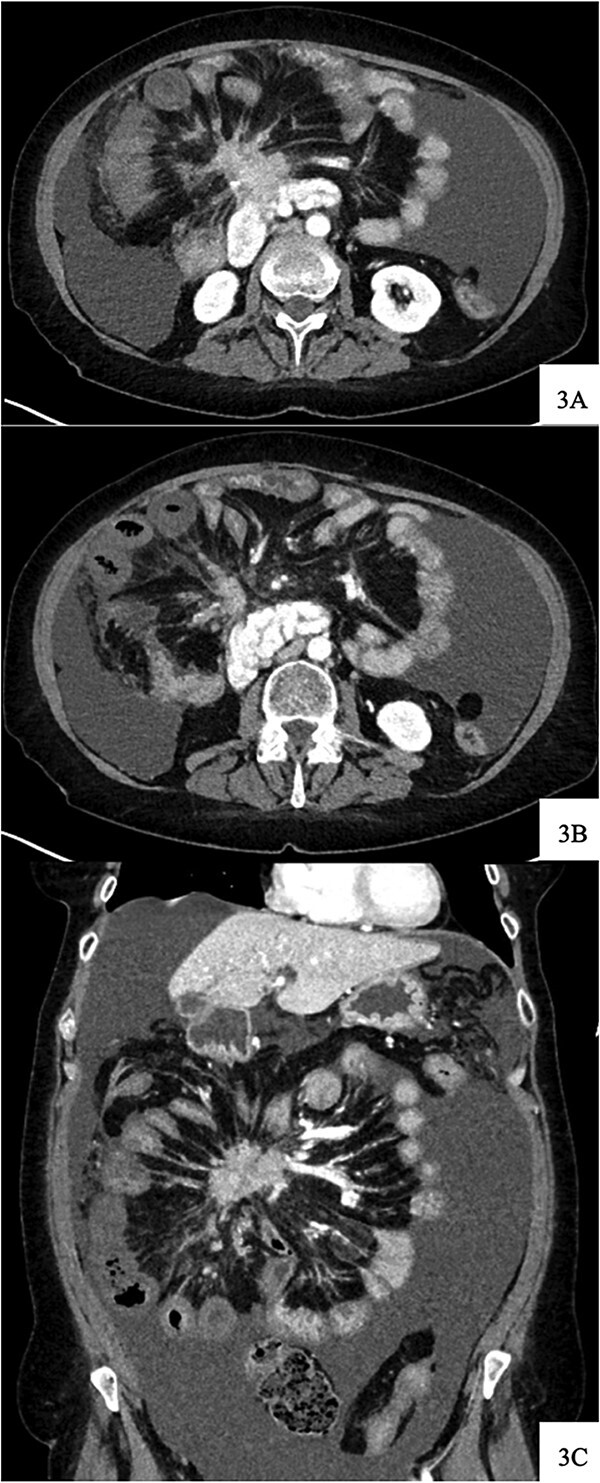
Contrast-enhanced axial CT image at time of presentation shows spiculated central mesenteric mass severe narrowing of the SMV with probably occlusion and encasement of the SMA with possible occlusion (A) associated with decreased enhancement and bowel wall thickening of several small bowel loops within the right lower quadrant (B). Coronal reformatting demonstrates the relationship of the mesenteric mass and affected small bowel (C).

Upon arrival, she was afebrile with stable vital signs but appeared uncomfortable with significant abdominal pain. Her labs revealed leukocytosis, anemia, and absent lactic acidosis. CT demonstrated decreased enhancement and bowel wall thickening of several small bowel loops within the right lower quadrant, which were new compared to prior imaging (Fig. 4).

Given her abrupt-onset abdominal pain in the setting of a tumor involvement of the SMA and imaging findings concerning for bowel ischemia, the decision was made to proceed with exploratory laparotomy after discussion regarding goals of care with the patient and her family.

Upon entry into the abdomen, she was found to have chylous ascites and a foreshortened mesentery adherent to her large mesenteric mass. A 40 cm, nonviable loop of intestine was identified 10 cm from the ileocecal valve. A small bowel resection was performed, and she was left in discontinuity with temporary abdominal closure with plan for second-look surgery in 48 hours.

Upon return to the operating room, all remaining bowel appeared viable. An additional 10 cm of ileum was resected proximally to ensure a healthy, tension-free anastomosis. An ileocecectomy was performed; however, her right colon could not be mobilized due to significant tethering to the mass. An ileocolic anastomosis was successfully created, and the middle colic pedicle as well as the small bowel mesentery at the site of the anastomosis had doppler signals at the end of the case.

Final pathology revealed viable small bowel margins that were negative for dysplasia or malignancy. This patient’s post-operative course was complicated by Klebsiella bacteremia but was otherwise unremarkable, and she recovered appropriately.

## Discussion

Acute-onset abdominal pain in a patient with NET warrants urgent. Distinguishing between pain resulting from obstruction versus that arising from bowel ischemia may be challenging due to similarities in clinical presentations in the early stages. Mesenteric ischemia, while rare, should not be overlooked, as timely diagnosis and intervention are imperative.

The pathogenesis of mesenteric ischemia secondary to SBNETs has been a point of controversy for many decades. The initial documentation of this association dates back to 1959 and attributes the finding to mass effect from mesenteric nodules inhibiting venous outflow [6]. Later, a fibrotic reaction in the mesenteric root was thought to cause extraluminal pressure to mesenteric vasculature [4, 7]. The idea that hormones secreted from the tumor, particularly 5-hydroxytryptamine, subsequently came about, bringing two new ideas: elastic sclerosis and vasospasm [8–11]. Obliterative elastic sclerosis refers to intraluminal narrowing and obstruction from proliferation of elastic tissue in the adventitia prompted by serotonin [12]. Vasospasm, contrarily, is secondary to direct effect of serotonin on the smooth muscle cells and fibroblasts of the vessel wall causing spasmodic contraction and intermittent vessel occlusion [11]. Others attributed ischemia to statis and thrombus formation as a result of a combination of the previously mentioned factors [13]. An extensive review of the topic done in 2004 could not definitively conclude the pathogenesis of NET-associated fibrosis [14].

Treatment of mesenteric ischemia with complete oncologic resection in additional to ischemic bowel resection is optimal. However, in patients with advanced, unresectable disease, such as this patient, complete oncologic resection may compromise the main mesenteric vessels, resulting in worsening ischemia and short bowel syndrome. Therefore, limited resection of the ischemic bowel with careful dissection, preservation of threatened bowel, and second-look laparotomy may be necessary [15]. Bypass procedures are typically avoided as continued tumor growth may prompt ischemia in a disengaged segment. In patients with acute-on-chronic mesenteric ischemia, collateralization may mitigate the need for a more extensive resection.

## Conflict of interest statement

None declared.

## Funding

None declared.
